# Advancing Prostate Cancer Staging: A Single-Step Approach With Bi-parametric and Whole-Body Diffusion MRI in an African Cohort

**DOI:** 10.7759/cureus.59470

**Published:** 2024-05-01

**Authors:** George Asafu Adjaye Frimpong, Evans Aboagye, Emmanuel Asante, Kwaku Addai A Appiah, Osei Owusu-Afriyie, Adwoa O Asare, Dorcas Atuobi, Bernard D Akpaloo, Bright Antwi

**Affiliations:** 1 Radiology, Kwame Nkrumah University of Science and Technology, Kumasi, GHA; 2 Radiology, Spectra Health Imaging and Interventional Radiology, Kumasi, GHA; 3 Research and Development, Spectra Health Imaging and Interventional Radiology, Kumasi, GHA; 4 Surgery, Kwame Nkrumah University of Science and Technology, Kumasi, GHA; 5 Pathology, Kwame Nkrumah University of Science and Technology, Kumasi, GHA; 6 Oncology, Komfo Anokye Teaching Hospital, Kumasi, GHA

**Keywords:** african men, staging, whole-body diffusion-weighted mri, biparametric mri, prostate cancer

## Abstract

Objectives: To document our initial experience using whole-body diffusion-weighted magnetic resonance imaging (WB-DWI/MRI) and bi-parametric magnetic resonance imaging (bpMRI) as a single exam in the staging of biopsy-proven prostate cancers.

Methods: This retrospective study involved 120 African men with biopsy-confirmed prostate cancer (PCa). All the participants had a single exam that included both a bpMRI and a WB-DWI/MRI. The results were analyzed based on the American Urological Association's risk stratification system and evaluated using descriptive statistics.

Results: The combined imaging approach confirmed PCa in all cases, identifying pelvic lymph node metastases in 21 (17.5%) patients. Among 72 high-risk patients, bpMRI+WB-DWI/MRI detected pelvic lymph node metastases in 18 (25.0%), bone metastases in 15 (20.8%), retroperitoneal lymph node metastases in six (8.3%), and extraprostatic extension in 18 (25%), with no solid organ metastases observed.

Conclusion: The combination of WB-DWI/MRI and bpMRI in a single-step approach demonstrates diagnostic potential in primary prostate cancer staging for high-risk groups, with the added advantage of shorter examination times, lower patients’ costs, and elimination of the risks of adverse events associated with the use of contrast agents and exposure to radiation.

## Introduction

Prostate cancer (PCa) remains a leading cause of morbidity and mortality among men worldwide, with its prevalence and impact being particularly pronounced in African male populations [1]. Early and accurate staging is crucial for determining the most appropriate treatment strategies, thereby improving patient outcomes. With an emphasis on clinical staging, the use of imaging provides important information on the location, size of the lesion, and extent of the spread of the disease, which may be limited by traditional diagnostics like prostate-specific antigen (PSA) tests, nomograms, transrectal ultrasound-guided biopsy (TRUS), and digital rectal examination [2].

Multiparametric MRI (mpMRI) is currently the recommended imaging modality for the local staging of PCa. It employs a comprehensive diagnostic approach incorporating T2-weighted imaging (T2WI), diffusion-weighted imaging (DWI), and dynamic contrast enhancement sequences (DCE). Despite the numerous advantages of mpMRI, limitations such as its high cost, use of gadolinium-based contrast agents, and longer time required to complete studies undermine its application [3]. Recently, bi-parametric MRI (bpMRI) has emerged as an alternative to mpMRI, incorporating the same sequences. However, the exclusion of the DCE sequence eliminates the risks of adverse events associated with the use of contrast agents and reduces examination time [4-6]. Several studies have concluded that bpMRI has a similar diagnostic performance as mpMRI for detecting prostate cancer [7,8], with excellent inter-reader agreement [9,10].

The American Urological Association (AUA) and European Urology Association (EUA) recommend Positron Emission Tomography/Computed Tomography (PET/CT) as the preferred imaging modality for detecting prostate cancer metastases. PET/CT offers superior diagnostic accuracy over bone scans (BS) and CT. However, for institutions that do not have access to PET/CT, whole-body diffusion-weighted MRI (WB-DWI/MRI) remains a powerful alternative with proven superiority over BS and CT for the detection of distant metastasis and evaluation of disease response to treatment [11]. WB-DWI/MRI evaluates bone and soft tissue disease and shows cell viability and tissue cellularity, thus increasing confidence in detecting, characterizing, and monitoring treatment response in metastatic lesions [11,12]. Additionally, the apparent diffusion coefficient (ADC) map obtained from diffusion-weighted MRI reflects cellular density, tumor morphology, nuclear-to-cytoplasm ratio, and integrity of cell membrane, regardless of cancer type and location [13,14].

This study shows our first experience with a protocol that combines WB-DWI/MRI and bpMRI in a single step for the staging and monitoring of African men with prostate cancer that has been confirmed by biopsy but has not yet received treatment. The focus is on documenting the practical aspects, outcomes, and potential advantages of integrating advanced MRI techniques in the clinical setting.

## Materials and methods

The study received ethical approval from the Committee on Human Research, Publication, and Ethics at Kwame Nkrumah University of Science and Technology, Kumasi, Ghana (CHRPE/RC/028/20). This retrospective study included 120 biopsy-proven prostate cancer patients who underwent a single-step examination that combined WB-DWI/MRI and bpMRI for suspected localized and metastatic disease from January 2020 to December 2022. The treating doctors referred patients to our facility because they had a positive prostate biopsy, an elevated PSA, and signs of metastatic disease. Demographic and clinical parameters, including the PSA and Gleason score, were collected for each patient. Patients already treated for PCa and those undergoing treatment were excluded from the study. All imaging studies were done within three months after the biopsy and between 2018 and 2022. Patients were categorized into three groups based on their risk assessment: low-risk (PSA <10 and Gleason grade group 1 and clinical stage T1-T2a), intermediate risk (Gleason grade group 2-3, and/or PSA 10-20 ng/ml, and cT≤2b), and high-risk (Gleason grade group 4, and/or PSA >20 ng/ml, and/or cT≥2c) [15].

MRI examination

All images were acquired on a Siemens Magnetom Essenza 1.5 Tesla 16-Channel TIM MRI scanner (Siemens, Germany). The prostate examination had the following sequences: High-resolution T2 images in axial, coronal, and sagittal planes and axial DWI (B50-1400) with ADC mapping. The axial DWI examination was performed using the Siemens proprietary high-resolution diffusion sequence RESOLVE. Fusion of the axial T2 and axial diffusion images with color coding for improved visualization was employed. The WB-DWI/MRI and bpMRI sequences used in the study are presented in Table 1 and Table 2, respectively.

**Table 1:** Sequences used in WB-DWI/MRI STIR: Short tau inversion recovery, DWIBS: Diffusion-weighted whole-body imaging with background suppression, TR/TE: Repetition Time/Time to Echo, FOV: Field of view, TSE: Turbo spin echo.

Parameters	DWIBS	STIR TSE	T1W TSE	T2W TSE
Imaged area	Neck-Pelvis	Neck-Pelvis	Neck-Pelvis	Neck-Pelvis
Plane	Axial	Sagittal	Sagittal	Axial
TR/TE (msec)	14200/850	3200/40	550/18.0	2500/92
Flip angle (deg)	-	150	150	160
FOV (mm)	400	300	300	380
Matrix	1.6 x 1.6 x 5.0	0.6 x 0.6 x 4.0	0.6 x 0.6 x 4.0	1.5 x 1.5 x 6.0
Slice thickness (mm)	5.0	4.0	4.0	6.0
Scan techniques	IR	TSE	TSE	TSE
Fat suppression	-	-	-	-
b-factors (s/mm^2^)	B-900 1/50	-	-	-
Number of sections	3	3	3	3
Acquisition time (one section)	5:50	2:06	1:01	1:00

**Table 2:** Sequences used in bpMRI bpMRI: Bi-parametric magnetic resonance imaging, tse: Turbo spin echo, tra: Transversal.

Sequence	Region	Plane of acquisition	Time of repetition (ms)	Time of echo (ms)	Slice thickness (mm)	Voxel size/reconstructed (mm x mm x mm)	Field of view (mm x mm)	Scan time (min: sec)
T2 Axial	Prostate	Axial	3500.0	108	2.2	0.3x0.3x2.2	160	5.45
T2 Coronal	Prostate	Coronal	7500.0	109	3.0	0.5x0.5x3.0	170	5.09
T2 Sagittal	Prostate	Sagittal	3500.0	108	2.2	0.3x0.3x2.2	160	5.45
Resolve_diffusion b50_1400_tra	Prostate	Transversal	7180.0	80	4.0	2.0x2.0x4.0	230	6.08

The imaging results were based on a review of original findings from reports by consulting radiologists who specialize in oncological imaging. The process involved two radiologists with 10 and 15 years of experience in prostate MRI. For this study, metastatic disease is that which is identified on the whole-body MRI. The definition of metastasis was at the discretion of the reporting radiologist and was descriptive, not adhering to a standardized scheme. Patients were classified as having metastases if they had a low or iso-signal on T2W compared to muscle tissue and a high signal on DWI and STIR. As seen on WB-DWI/MRI, metastases were divided into three groups: bone metastases, pelvic lymph node metastases, and retroperitoneal lymph node metastases. The presence or absence of extraprostatic extension was recorded.

Statistical analysis

The patient demographics and baseline clinical values among study participants were described. Based on the initial MRI data, a descriptive definition of metastasis was used. The site of metastases was registered, and the results were classified as either positive or negative for extraprostatic extension, lymph, and bone metastasis. A descriptive analysis of the frequency of extraprostatic extension, bone, pelvic lymph node, and retroperitoneal lymph node metastases was conducted. All analyses were performed on SPSS software, version 27 (IBM Corp., Armonk, NY).

## Results

Patient demographics and clinical characteristics

A total of 120 patients were included in the study. Table 3 presents the patient demographics and clinical characteristics of the study population. The mean age of the patients was 67 years, with a standard deviation of 8.45. The majority of the patients were in the age group of 60-69 years (47.5%), followed by the age groups of 70-102 years (39.2%) and 35-59 years (13.3%). The median PSA was 23.9 ng/ml, with an interquartile range (IQR) of 13.48-67.04 ng/ml. The Gleason score (GS), which indicates the aggressiveness of prostate cancer, varied among the patients. GS 10 (n = 1, 0.8%), GS 6 (n = 17, 14.2%), GS 9 (n = 19, 15.8%), and GS 8 (n = 24, 20.0%) were next in frequency to GS 7 (n = 59, 49.2%), which was the most prevalent. According to the risk assessment, 72 (60.0%) of the patients were classified as high-risk, 41 (34.2%) as intermediate-risk, and seven (5.8%) as low-risk. Regarding lesion type, the majority of patients had multiple lesions: 55 (45.8%), then single lesions: 44 (36.7%), and double lesions: 21 (17.5%). The left peripheral zone, with 72 (60.0%) cases, was the most common location of lesions, followed by 67 (55.8%) with lesions in the right peripheral zone. Lesions in the transitional zones were also observed, with 51 (42.5%) cases having lesions in the left transitional zone and 51 (41.7%) having lesions in the right transitional zone. Additionally, 17 (14.2%) cases were found with anterior fibromuscular stroma lesions, 10 (8.3%) with central gland lesions, and six (5.0%) cases with diffused lesions.

**Table 3:** Patient demographics and clinical parameters

Variable	Frequency (n=120)
Age (Years) (π±SD)	67 ±8.45
Age Group (Years)	-
35-59	16 (13.3%)
60-69	57 (47.5%)
70-102	47 (39.2%)
PSA (ng/ml) [(Median (IQR)]	23.9 (13.48-67.04)
Gleason Score	No.
6	17 (14.2%)
7	59 (49.2%)
8	24 (20.0%)
9	19 (15.8%)
10	1 (0.8%)
Risk assessment	-
Low risk	7 (5.8%)
Intermediate risk	41 (34.2%)
High risk	72 (60.0%)
Lesion type	-
Single	44 (36.7%)
Double	21 (17.5%)
Multiple	55 (45.8%)
Location of Lesion	-
Right peripheral zone	67 (55.8%)
Left peripheral zone	72 (60.0%)
Right transitional zone	50 (41.7%)
Left transitional zone	51 (42.5%)
Anterior fibromuscular stroma	17 (14.2%)
Central gland	10 (8.3%)
Diffuse	6 (5.0%)

**Figure 1:**
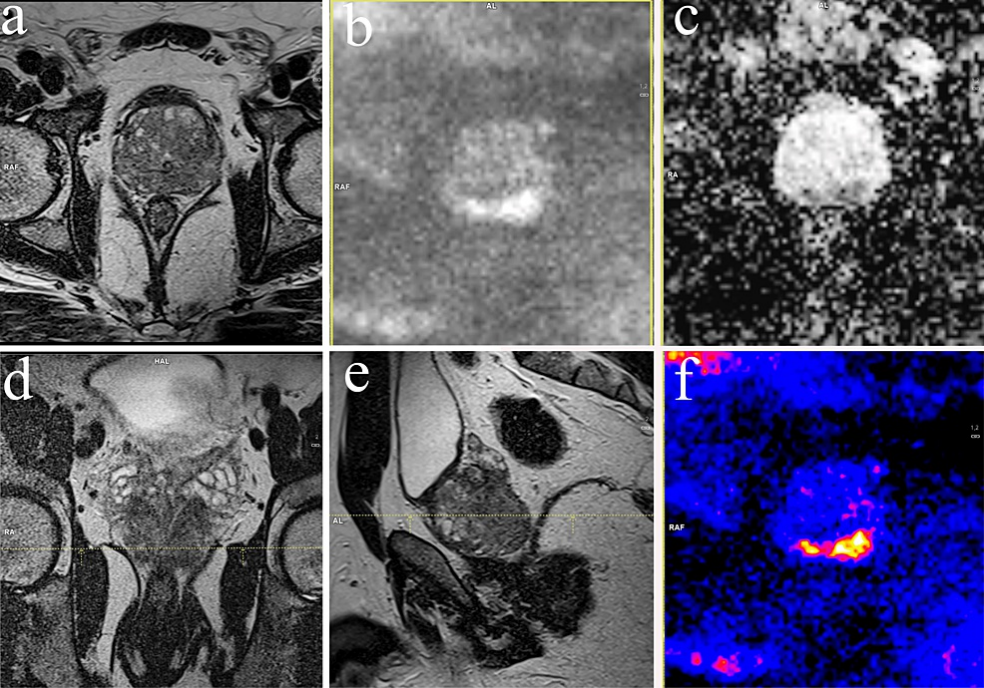
Bi-parametric imaging of the prostate Bi-parametric imaging of the prostate depicting axial, sagittal, and coronal T2 images with color coding (a, d, e, and f) and axial diffusion and apparent diffusion coefficient (ADC) map (b and c). bpMRI shows bilateral, peripheral malignant lesions appearing as ill-defined hypointense on T2 sequences. They show restriction in the diffusion sequence and appear hypointense in the ADC map.

**Figure 2:**
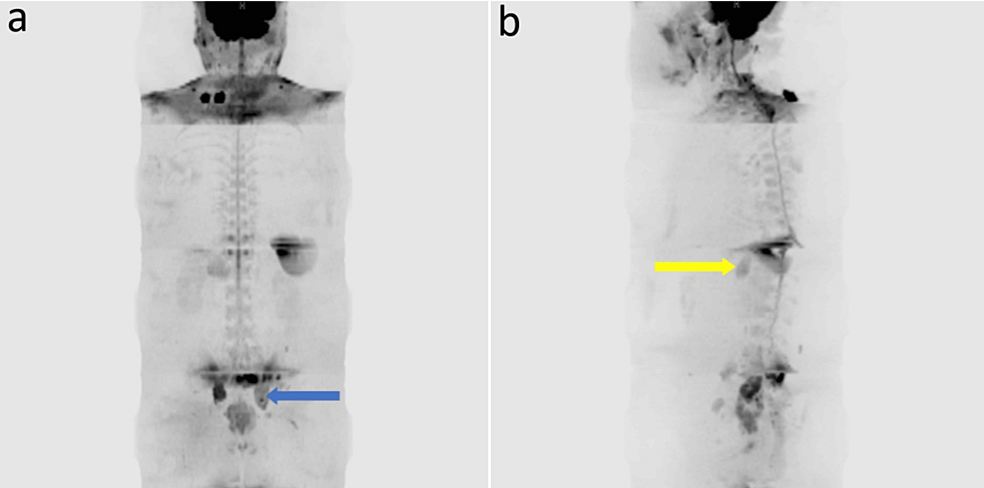
WB-DWI/MRI with coronal MIP reconstruction Whole-body diffusion-weighted MRI (WB-DWI/MRI) with coronal maximum intensity projection (MIP) reconstruction showing metastatic prostate cancer, with (a) pelvic bone metastasis (blue arrow) and (b) retroperitoneal metastases (yellow arrow).

Figure 1 and Figure 2 show localized and metastatic disease assessment on bpMRI and WB-DWI/MRI, respectively.

Table 4 presents the bpMRI+WB-DWI/MRI findings according to risk group stratifications. In the low-risk group, no cases of extraprostatic extension, bone metastases, pelvic lymph node metastases, or retroperitoneal lymph node metastases were detected. Among the intermediate-risk group, extraprostatic extension was observed in four (9.8%) cases, bone metastases in one (2.4%), pelvic lymph node metastases in three (7.3%), and no retroperitoneal lymph node metastases were identified. In the high-risk group, extraprostatic extension was present in 18 (25.0%) cases, bone metastases in 15 (20.8%) cases, pelvic lymph node metastases in 18 (25.0%) cases, and retroperitoneal lymph node metastases in six (8.3%) cases.

**Table 4:** WB-DWI/MRI and bpMRI findings according to risk group stratification WB-DWI/MRI: Whole-body diffusion-weighted magnetic resonance imaging, bpMRI: Bi-parametric magnetic resonance imaging.

Risk stratification (n=120)	Extraprostatic extension	Bone Metastases	Pelvic lymph node metastases	Retroperitoneal lymph node metastases
Low risk (n=7)	0 (0.0%)	0 (0.0%)	0 (0.0%)	0 (0.0%)
Intermediate risk (n=41)	4 (9.8%)	1 (2.4%)	3 (7.3%)	0 (0.0%)
High risk (n=72)	18 (25.0%)	15 (20.8%)	18 (25.0%)	6 (8.3%)

## Discussion

To the best of our knowledge, this is the first study to introduce a protocol that combines bpMRI and WB-DWI/MRI in a single session for staging primary prostate cancer in a high-risk population. The closest reported protocol used multi-parametric MRI (mpMRI), which was used to assess disease recurrence and metastatic disease after radical prostatectomy [16]. Despite the absence of DCE, results from recent studies have demonstrated that bpMRI and mpMRI perform comparably in terms of detecting prostate cancer [7,8]. Whole-body MRI is also considered a more accurate test for detecting bone disease than conventional bone scintigraphy (BS) and CT [17-20]. Therefore, leveraging the known capabilities of these modalities, we combined bpMRI and WB-MRI to provide incremental information for staging biopsy-proven prostate cancers in a high-risk African population.

In this present study, the reported protocol was successful in confirming localized disease in all 120 biopsy-proven prostate cancer cases, further emphasizing the potential role imaging studies can play in the early detection of the disease. This study’s use of AUA's risk stratifications provides a background to understand primary staging findings on MRI vis-à-vis the nature of patients included in the study. It is also of great importance to note that AUA’s 2020 Policy Statement and the National Comprehensive Cancer Network’s (NCCN) updated guidelines have recommended abdominal and pelvic imaging for intermediate, high, and very high-risk PCa groups for disease assessment [15,21].

Traditionally, CT has been indicated for lymph node assessment, but previous studies have reported its limited ability to detect small-volume, micrometastatic nodal disease [22]. Lymph node detection on MRI is limited to evaluations including size >8 mm, rounded shape, loss of fatty hilum, irregular border, and low T2W signal relative to the primary tumor [23]. Diffusion-weighted imaging (DWI) can detect cellular and microstructural alterations in malignant lymph nodes, which can be quantified by the calculation of the ADC [22]. The DWI sequence in bpMRI augments the detection rate of lymph node metastases, which otherwise would be limited to lesion size and shape. The protocol detected lymph node metastases in 27 cases (21 pelvic and six retroperitoneal), making it the most detected metastatic site. Interestingly, it was also highest in both high-risk and intermediate-risk groups. This study concurs with others that have shown pelvic lymph nodes to be a prominent site and route of prostate cancer spread to other metastatic sites [24].

Several studies have reported the superiority of WB-DWI to BS in detecting bone metastases. The meta-analysis study by Shen et al. [25] looked at how well choline-PET/CT, BS, single-photon emission computed tomography, and WB-MRI could find metastasized (M1b) prostate cancer lesions. Compared to BS, which had a pooled sensitivity of 0.82 (95% CI: 0.78-0.85), WB-MRI showed higher sensitivity at 0.95 (95% CI: 0.90-0.97) on a per-lesion basis [25]. Our study identified bone metastases in 16 cases (13.3%), with 14 cases in the high-risk group and two in the intermediate-risk group. Our findings are consistent with studies that have shown that bones are common sites for prostate cancer spread [26].

The occurrence of extraprostatic extension (EPE) is mostly associated with aggressive diseases, emphasizing the need for its evaluation during the local staging of prostate cancer [27]. EPE is typically assessed by considering clinical factors like PSA values, Digital Rectal Examination, and Gleason scores at biopsy [28]. These techniques, however, without MRI, fall short and provide no details regarding the location and scope of EPE [29]. According to Guerra et al. [30], EPE that can be measured using MRI is highly correlated with its presence in pathology. In our study, the prevalence of MRI-detected EPE was 22 (18.3%), of which the majority were found in the high-risk group. This finding aligns with the AUA risk assessment [15], which recognizes patients with EPE as high-risk. bpMRI did not identify EPE in the low-risk group and again agrees with the AUA risk assessment. However, four intermediate-risk patients recorded EPE, which emphasizes the role of imaging in correctly placing patients in appropriate risk assessment groups.

This MRI-based study is not without limitations. Firstly, the study is retrospective and was done at a single centre, although a major referral site. Secondly, the specific subtypes of prostate cancer imaged were not delineated in the study. Furthermore, the study leveraged on already established capabilities of bpMRI and WB-DWI/MRI in staging PCa and did not compare metastases on MRI to histopathology or other imaging modalities. Future studies are warranted to validate the use of bpMRI + WB-DWI/MRI as a single examination in other high-risk populations.

## Conclusions

This study introduces a protocol for the primary staging of PCa, involving a combination of WB-DWI/MRI and bpMRI in a single-step approach. The protocol demonstrates diagnostic potential, with the advantage of shortening examination times, reducing hospital costs and visits, and eliminating the risks of adverse events associated with the use of contrast agents and radiation exposure. It also shows promising results for the effective evaluation of localized prostate disease, distant metastases, and extraprostatic extension to guide PCa treatment planning and prognosis. The findings of this study support the potential role of imaging studies, specifically WB-MRI and BP-MRI, in early detection, accurate staging, and individualized treatment planning for high-risk prostate cancer patients.
